# Combined Effect of Vitamin D Supplementation and Physiotherapy on Reducing Pain Among Adult Patients With Musculoskeletal Disorders: A Quasi-Experimental Clinical Trial

**DOI:** 10.3389/fnut.2021.717473

**Published:** 2021-10-05

**Authors:** Mohammad Ali, Zakir Uddin, Ahmed Hossain

**Affiliations:** ^1^Department of Physiotherapy and Rehabilitation, Uttara Adhunik Medical College and Hospital, Dhaka, Bangladesh; ^2^Hasna Hena Pain, Physiotherapy and Public Health Research Center, Dhaka, Bangladesh; ^3^School of Rehabilitation Sciences, McMaster University, Hamilton, ON, Canada; ^4^Department of Public Health, North South University, Basundhara, Dhaka, Bangladesh; ^5^North South University (NSU) Global Health Institute, Basundhara, Dhaka, Bangladesh

**Keywords:** clinical trial, combined therapy, musculoskeletal disorder, physiotherapy, vitamin D, quasi-experimental design

## Abstract

**Background:** The prevalence of musculoskeletal disorders (MSD) and vitamin D insufficiency is high. Past research indicating vitamin D supplementation and physiotherapy are useful for the treatment of MSDs. However, to the best of our knowledge, the combined benefits of vitamin D and physiotherapy are not yet evaluated in MSD. We hypothesized that combined intervention of vitamin D supplementation and physiotherapy would be more effective in relieving the pain of MSD compared to only physiotherapy intervention.

**Objective:** This study aimed to examine whether combined effect of vitamin D supplementation and physiotherapy was better than physiotherapy alone in reducing pain-related outcomes (e.g., pain severity, affective interference, and physical interference) in patients with MSD.

**Methods:** A quasi-clinical experiment was conducted between May 2020 and April 2021. Vitamin D level in the blood was measured at the start of the study. Patients with vitamin D levels <20 ng/mL were assigned to the combined physiotherapy and vitamin D group. The physiotherapy group consisted of the patients with vitamin D levels more than 20 ng/mL. The Brief Pain Inventory scale was utilized to measure pain at baseline and after 12 weeks of interventions. We used the paired *t*-tests for unadjusted analysis. Further, a linear regression model was used to identify the combined effect of physiotherapy and vitamin D on reducing pain scores after adjusting potential confounders.

**Results:** Combined intervention of vitamin D and physiotherapy showed significantly better results than only physiotherapy intervention in the reduction of three pain-related outcomes (*p* < 0.001). The multivariable analysis indicated that the combination of physiotherapy and vitamin D treatment reduced pain score by 1.126 (slope = −1.126, *p* = 0.035) compared to physiotherapy alone.

**Conclusion:** Combined intervention (vitamin D with physiotherapy) had a better pain-relieving effect than physiotherapy alone. To confirm these findings, more research is needed with randomized control trials.

**Clinical Trial Registration:** [http://ctri.nic.in/Clinicaltrials/advancesearchmain.php], identifier [CTRI/2020/04/024845].

## Introduction

According to the Global Burden of Disease study 2019, 1.7 billion people worldwide exhibit musculoskeletal disorders (MSD), which is the leading cause of disability (1). Low back pain (LBP) is the most common symptom of MSD, followed by neck pain, fractures, traumas, and osteoarthritis. The global point prevalence of activity-limiting single MSD (i.e., LBP) in 2015 was 7.3%, implying that 540 million people were affected by LBP at any one moment of life (2).

Furthermore, the world population has a significant frequency of serum 25-hydroxyvitamin D (25(OH) D) (vitamin D) deficiency. Vitamin D deficiency affects 24% Americans, 37% Canadians, and 40% Europeans; in India, 490 million people exhibit vitamin D deficiency (3). Vitamin D deficiency can affect up to 99% of people depending on their age and ailments (3). In Bangladesh, the incidence of vitamin D deficiency among various groups of individuals is as high as 100% (4–6).

A positive association between vitamin D deficiency and the leading MSD, such as LBP, neck pain, and knee pain, among others has been indicated. A triple-arm controlled study suggested that severe vitamin D deficiency is associated with sub-acute and chronic LBP (7). A systematic review and meta-analysis of observational studies concluded that vitamin D deficiency is significantly associated with LBP (8). Other studies also revealed the association between vitamin D deficiency and neck pain (9, 10). Furthermore, there is a remarkable association between vitamin D deficiency and knee pain (11). In general, the association between vitamin D deficiency and MSD (e.g., arthritis, muscle pain, and chronic widespread pain) is well-known (12).

Despite having a variety of options, managing MSD can be difficult and expensive; nonetheless, the results of any given treatment are not always adequate and are not recommended for universal usage (13, 14). Supplementation of vitamin D is a cost-effective treatment option for patients with MSD and a systematic review summarized that vitamin D supplementation can decrease pain scores and improve pain; however, no statistically significant change in the visual analog scale of pain intensity has been observed (15). On the other hand, physiotherapy interventions have frequently been used for treating MSD conditions, such as LBP, neck pain, shoulder pain, and knee pain, and numerous studies found physiotherapy interventions effective (16, 17). Nonetheless, it is hypothesized that the combination of both physiotherapy interventions and vitamin D supplementation can be more effective for patients with MSD than a single intervention. To the best of our knowledge, no study has examined the combined effect of physiotherapy and vitamin D supplementation among patients with MSD. Therefore, the primary goal of this clinical investigation was to evaluate if vitamin D supplementation in addition to physiotherapy may significantly reduce pain intensity, affective interference, and physical interference in adult patients with MSD.

## Materials and Methods

### Study Participants

**Figure 1:**
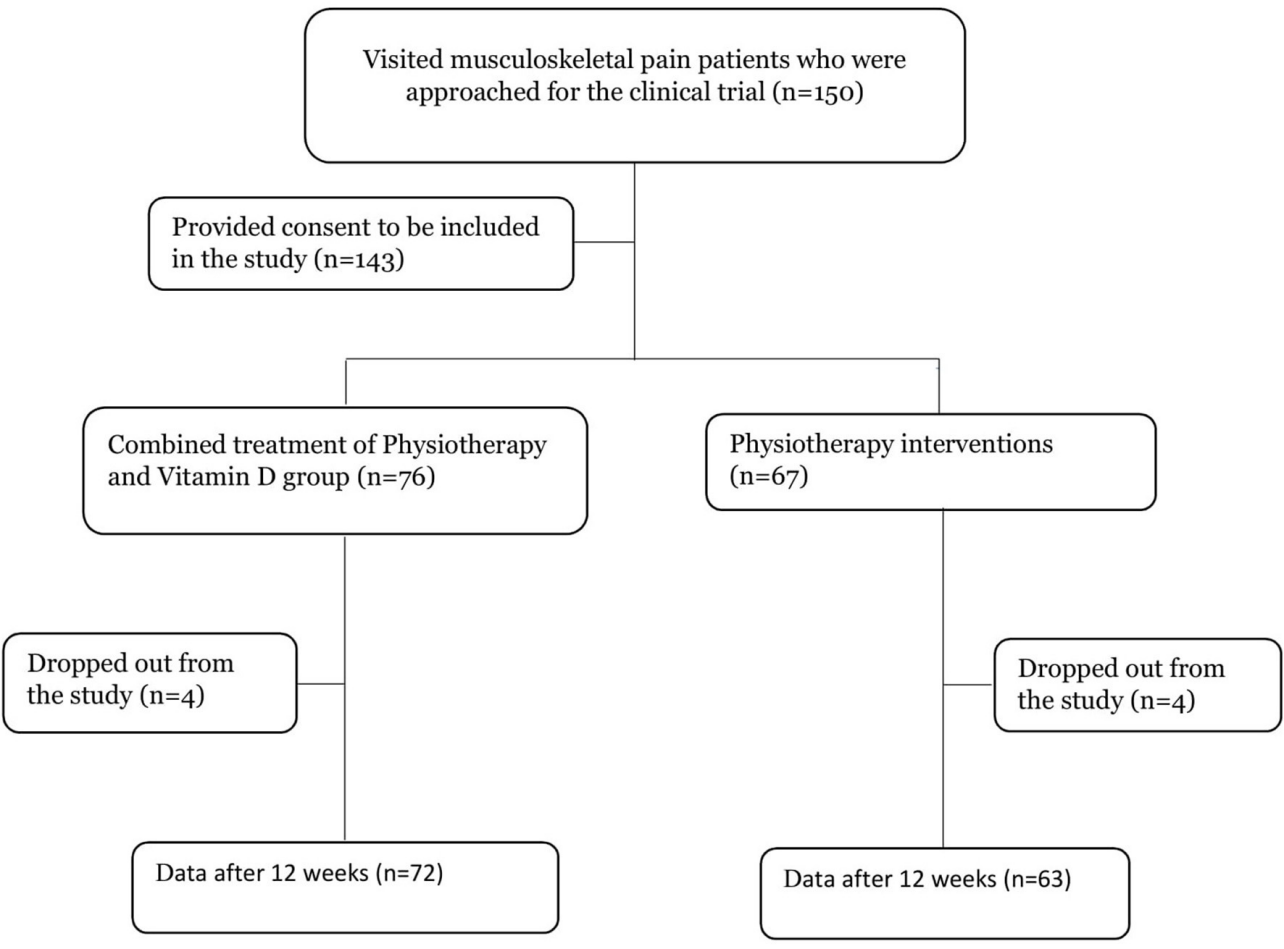
Flow chart of patients' selection.

Patients with musculoskeletal pain (LBP, neck pain, shoulder pain, and knee pain) aged 24–80 years who sought physiotherapy treatment at the physiotherapy and rehabilitation departments of Uttara Adhunik Medical College and Hospital in Dhaka and Hasna Hena Pain and Physiotherapy and Public Health Research Center in Dhaka city were randomly selected for this study. Patients under the age of 18 years and those who were experiencing pain because of cancer or tuberculosis were excluded from the study. Patients who did not adhere to the treatment plan or who desired to withdraw from the research were also excluded. Two hundred patients were eligible for the study based on the inclusion and exclusion criteria. We approached 150 people to ask whether they wanted to participate in the study, and 143 of them agreed. Eight patients later dropped out of the research. After 12-weeks, 135 patients finally gave data for the study. The flow chart has been presented in Figure 1.

### Study Design

This was a clinical trial using a quasi-experimental design. Data were collected at baseline and 12 weeks after the intervention between May 2020 and April 2021. Participants who gave their consent and met the inclusion criteria were interviewed using the Brief Pain Inventory (BPI) scale to record pain levels at baseline and after 12 weeks of pain relief intervention. Patients were also examined for vitamin D, hemoglobin, and nutrition levels. Following a standard protocol, a blood sample was collected from patients at the Uttara Adhunik Medical College and Hospital's Department of Pathology and Biochemistry.

### Measurements

#### BPI

##### Pain Severity

The pain severity subscale of BPI was used to measure self-reported overall pain severity. The pain severity subscale consists of four items that inquire for worst, least, and average pain intensity ratings within the last 24-h in addition to present pain ratings. Pain intensity is rated using an 11-point numerical rating scale in which 0 indicates “no pain” and 10 indicates “pain as bad as you can imagine.” An overall pain severity rating is calculated as a mean of the four items on pain intensity. The BPI is an internally consistent (Cronbach alpha = 0.82), reliable, and valid measure of pain severity and pain interference for people with musculoskeletal pain (18).

##### Pain Affective Interference

The BPI pain interference subscale consists of seven items that assess the extent to which pain interferes with the mood, relations with other people, and enjoyment of life (19). Responses are bounded by 0 (does not interfere) and 10 (interferes completely). Scores on pain affective interference were calculated as an average of their respective items.

##### Pain Physical Interference

Consistent with previous work, the physical interference subscale was calculated from BPI scale items relating to how pain interferes with a person's engagement in general activity, walking, and normal work-related activities (20). The three items in the subscale are designed to specifically quantify the degree to which pain interferes in activity engagement. Items are scored on an 11-point numerical rating scale (0–10), in which greater disruption to activity engagement due to pain is indicated by higher scores.

The physical and affective factor's items of BPI are supported by confirmatory factor and Rasch analysis (19, 20). Pain interference types (affective and physical) are helpful to guide clinical assessment in pain conditions (21–23). The BPI is widely used for people living with pain and is recommended for pain-related outcome measures (18–21, 24–26).

#### Nutritional Status

##### Mini Nutritional Assessment Scale

The mini nutritional assessment is a validated instrument initially developed to assess nutritional status in adult patients and is mainly indicated for research settings (27, 28). The tool contains 18 items and evaluates four different aspects: anthropometric assessment (body mass index, weight loss, and arm and calf circumferences); general assessment (lifestyle, medication, mobility, and presence of signs of depression or dementia); short dietary assessment (number of meals, food, and fluid intake and autonomy of feeding); and subjective assessment (self-perception of health and nutrition). After addition of the scores, labeled as *Mini Nutritional Assessment-Long Form*, individuals can be divided into three groups using threshold values of <17 for “malnourished,” 17–23.5 for “at risk of malnutrition,” and ≥24 for “normal nutritional status,” with a maximum total score of 30 points (29).

#### Vitamin D Level

Serum 25(OH)D was used to evaluate the vitamin D status among the study participants. The laboratory analysis was conducted at the Department of Biochemistry, Uttara Adhunik Medical College and Hospital. The serum concentration of 25(OH)D was measured by chemiluminescence microparticle immunoassay (ARCHITECT i 1000 SR, USA). Patients were classified based on vitamin D levels as deficient (<20 ng/mL); insufficient (20–30 ng/mL); and sufficient (>30–100 ng/mL) (6, 30).

#### Hemoglobin Level

A hematology analyzer (Sysmex XN-1000, Japan) was used to estimate the concentration of hemoglobin in the blood. Patients with hemoglobin levels between the ranges of 13–18 g/dL were considered normal.

### Patient Grouping and Interventions

The participants were distributed into two groups according to their vitamin D levels: physiotherapy and physiotherapy + vitamin D groups. The physiotherapy group consisted of patients who had vitamin D levels ≥ 20 ng/mL. Rest of the patients (vitamin D level < 20 ng/mL) were included in the physiotherapy + vitamin D group. All the patients were given traditional physiotherapy interventions for a maximum of 21 sessions. However, only the physiotherapy + vitamin D group was given a 40,000 IU vitamin D3 capsule made by a specific pharmaceutical company of Bangladesh per week for 8 weeks. To ensure the correct dose, patients were asked to visit the Uttara Adhunik Medical College and Hospital or Hasna Hena Pain and Physiotherapy and Public Health Research Center to take the capsule on full stomach at afternoon every week. A card with a chart of capsule-taking dates was maintaining to avoid any misconduct. Although there are no reported adverse effects of physiotherapy and vitamin D supplementation, the contact number of a physiotherapist and physician were given to the participants in case of any emergency or concern. Data were collected at the baseline and after 12 weeks.

### Ethics and Trial Registration

The study was conducted following the ethical standards outlined in the Helsinki Declaration (1983). The Ethics Review Committee of Uttara Adhunik Medical College and Hospital approved the study. This clinical trial was registered prospectively from World Health Organization endorsed Clinical Trials Registry CTRI/2020/04/024845, Registered on 24/04/2020, http://ctri.nic.in/Clinicaltrials/advancesearchmain.php. All participants provided written consent.

### Statistical Analysis

The acquired data were entered into the IBM SPSS 21 program (SPSS Inc., Chicago, IL, USA), and the statistical analysis was completed with R 3.6.3. The arithmetic mean and standard deviation (SD) were used to describe the continuous variables. Frequency and percentage were used to express categorical variables. For categorical variables, the chi-square and Fisher's exact tests were employed to determine the difference between the groups. Before and after the evaluation, the within-group comparisons were determined using a paired samples *t*-test. The results were assessed using a 95% confidence interval. Finally, after adjusting for sex and age group, a multivariable linear regression model was conducted to examine the combined effect of vitamin D and physiotherapy on change in pain scores.

## Results

This quasi-experimental study included 143 patients (mean ± SD age, 51.2 ± 13.3 years; range 24–80 years; 63.6% women). Table 1 provides the baseline results of the participants. At baseline, there were no significant differences between the two groups in terms of sex (*p* = 0.569), age (*p* = 0.065), body mass index (*p* = 0.125), exercise habit (*p* = 0.363), diabetes mellitus (*p* = 0.877), hypertension (*p* = 0.130), hemoglobin level (*p* =0.879), nutrition level (*p* = 0.537), main complaints (*p* = 0.166), and pain duration (*p* = 0.811).

**Table 1:** Baseline characteristics of the patients.

**Variable**	**All (%)**	**Interventions**		*p*-value^*^
		**Physiotherapy + Vitamin D (%)**	**Physiotherapy (%)**	
**Gender**				
Female	91 (63.6)	50 (54.9)	41 (45.1)	0.569
Male	52 (36.4)	26 (50)	26 (50)	
**Age group**				
≤ 40 years	38 (26.6)	22 (57.9)	16 (42.1)	0.065
41–60 years	66 (46.2)	40 (60.6)	26 (39.4)	
≥60 years	39 (27.2)	14 (35.9)	25 (64.1)	
**BMI group**				
Normal	56 (39.2)	24 (42.9)	32 (57.1)	0.125
Overweight/obese	87 (60.8)	52 (59.8)	35 (40.2)	
**Had walking/exercise habit**				
No	96 (67.1)	55 (57.3)	41 (42.7)	0.363
Yes	47 (32.9)	21 (44.7)	26 (55.3)	
**Diabetes group**				
No	108 (75.5)	57 (52.8)	51 (47.2)	0.877
Yes	35 (24.5)	19 (54.3)	16 (45.7)	
**Hypertension group**				
No	80 (55.9)	47 (58.8)	33 (41.3)	0.130
Yes	63 (44.1)	29 (46.0)	34 (54.0)	
**Hemoglobin level (g/dl)**				
Risk of anemia	74 (51.7)	42 (56.8)	32 (43.2)	0.879
Normal range	69 (48.3)	34 (49.3)	35 (50.7)	
**Nutrition level**				
Risk of malnourishment	78 (49.0)	39 (50.0)	39 (50.0)	0.537
Nourished	65 (54.5)	37 (56.9)	28 (43.1)	
**Main complaints**				
Low back pain	72 (50.3)	38 (52.8)	34 (47.2)	0.166
Neck pain	31 (21.7)	21 (67.7)	10 (32.3)	
Shoulder pain	16 (11.2)	8 (50.0)	8 (50.0)	
Knee pain	24 (16.8)	9 (37.5)	15 (62.5)	
**Pain duration**				
≤ 6 weeks	67 46.9)	32 (47.8)	35 (52.2)	0.099
7–11 weeks	8 (5.6)	7 (87.5)	1 (12.5)	
≥12 weeks	68 (47.6)	37 (54.4)	31 (45.6)	

* *p-value was calculated from chi-square test*.

**Figure 2:**
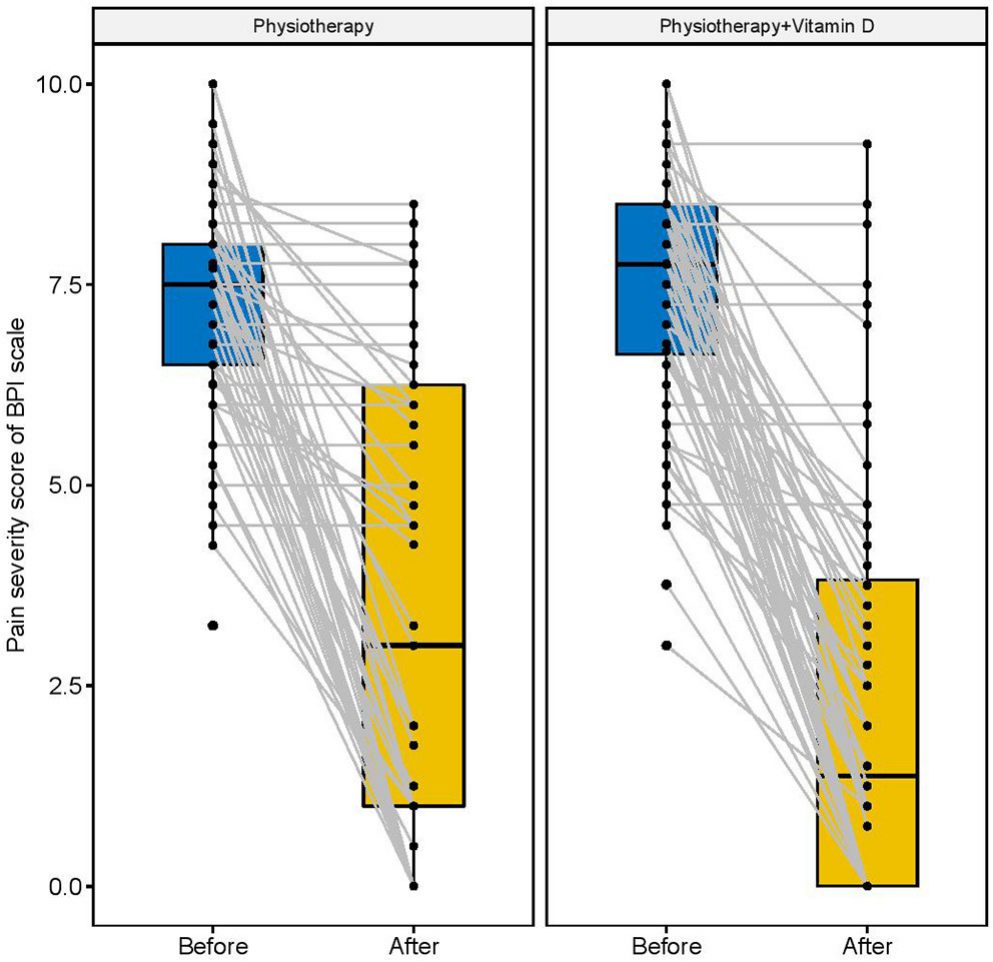
Pain intensity scores before and after intervention among both groups.

**Figure 3:**
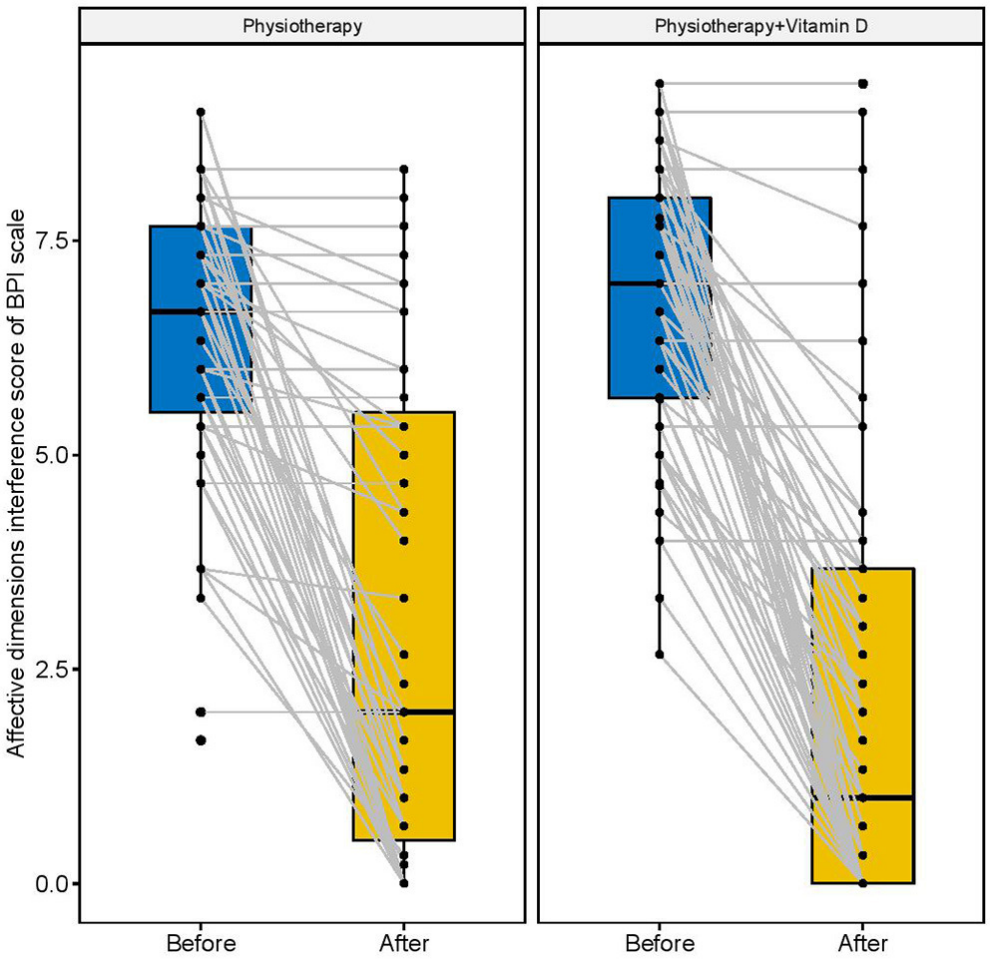
Physical interference scores before and after the intervention.

**Figure 4:**
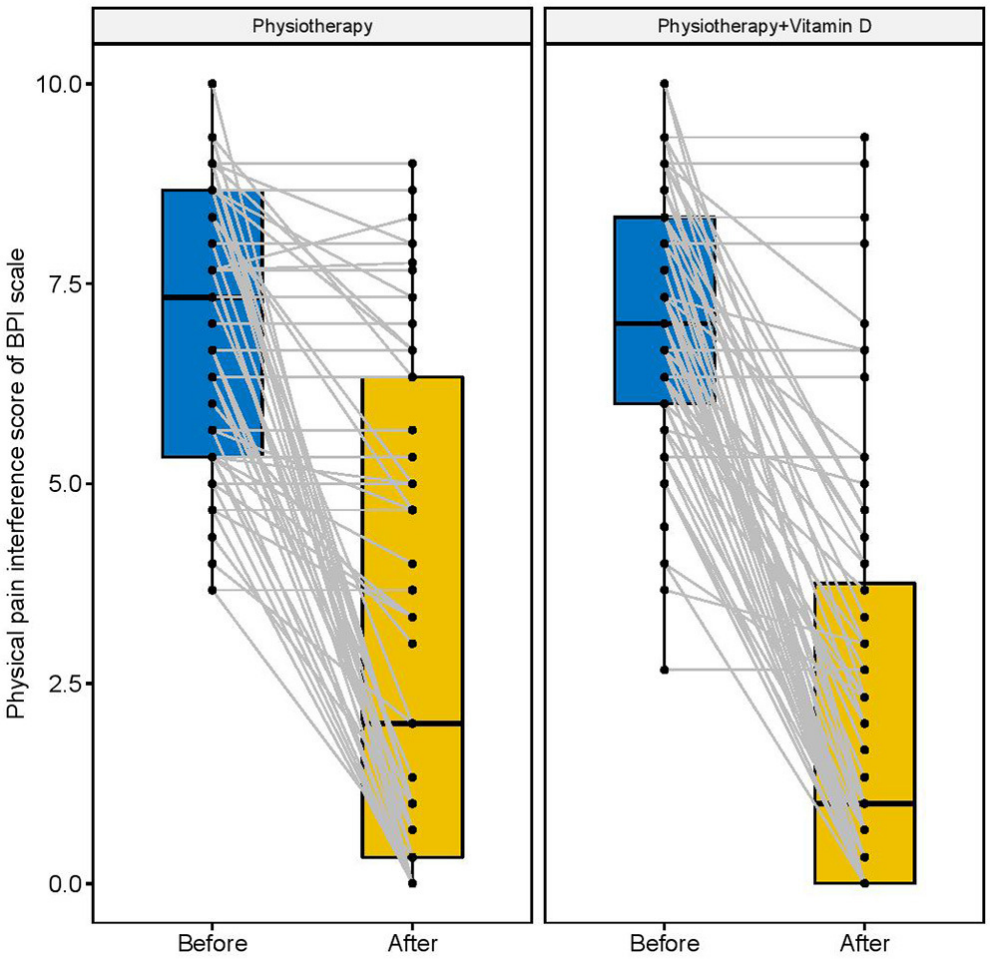
Affective interference scores before and after the intervention.

Figures 2–4 display the improvement in pain score after receiving the combination of vitamin D and physiotherapy treatment, and only physiotherapy intervention. In these figures (Figures 2–4) of pain severity, affective interference, and physical interference, the combination of vitamin D and physiotherapy treatment resulted in a significant reduction in pain scores compared to only physiotherapy intervention.

The descriptive results of changes in pain scores between before and after interventions are shown in Table 2. In comparison to the physiotherapy intervention, the combination of physiotherapy and vitamin D treatment resulted in a significant reduction in pain severity score (*p* = 0.001), affective interference score (*p* = 0.001), and physical interference score (*p* = 0.001).

**Table 2:** Change of pain score (mean difference before and after intervention) among physiotherapy + Vitamin D and Physiotherapy group after 12-week.

**Pain category**	**Physiotherapy** **+** **Vitamin D**		**Physiotherapy**		*p*-value^*^
	**Mean difference**	**95% CI**	**Mean difference**	**95% CI**	
Pain severity score	−4.92	−5.57, −4.26	−3.79	−4.58, −3.01	<0.001
Affective interference score	−4.63	−5.26, −3.99	−3.46	−4.20, −2.72	<0.001
Physical Interference score	−4.75	−5.36, −4.14	−3.80	−4.63, −2.96	<0.001

*CI, Confidence Interval*.

* *p-value was calculated from the t-test*.

The findings of a multivariable linear regression model adjusted for sex and age are presented in Table 3. The findings revealed that age and sex had no effect on the reduction of overall pain in patients with MSD. When compared to physiotherapy intervention alone, the combination of physiotherapy and vitamin D treatment reduced pain score by 1.126 (slope = −1.126, *p* = 0.035).

**Table 3:** Multivariable linear regression analysis on an overall change of pain score.

**Variable**	**Slope of change score**	**Standard error**	***p*-value**
**Gender**			
Female	Reference		
Male	0.466	0.64	0.391
**Age group**			
≤ 40	Reference		
41–60	−0.226	0.63	0.719
≥60	−0.452	0.71	0.525
**Intervention group**			
Physiotherapy	Reference		
Physiotherapy + Vitamin D	−1.126	0.53	0.035^*^

* *Significant at 5% significance level*.

## Discussion

The study shows irrespective of all measured potential confounders, vitamin D3 supplementation in addition to physiotherapy was significantly effective in reduction of pain severity and affective and physical interface. This novel finding provides a scientific basis on the role of vitamin D in improving musculoskeletal pain outcomes.

To the best of our knowledge, we provide here the first quasi-experimental data of patients with musculoskeletal pain examining the effect of vitamin D supplementation in addition to physiotherapy compared to only physiotherapy intervention on the reduction of pain parameters (e.g., pain severity, affective pain interference, and physical pain interference). In line with our findings, a systematic review and meta-analysis of published randomized controlled trials suggested that vitamin D supplementation can decrease pain scores significantly (31). Another review also concluded that vitamin D may establish a safe, simple, and potentially beneficial way to reduce pain among patients with vitamin D deficiency (32). However, systematic reviews presented evidence that high vitamin D concentration in the blood is associated with good cognitive function in adults (33, 34). Regarding physical interference, systematic reviews found inconclusive results (35, 36). Previous evidence of the positive impact of physiotherapy on physical activity promotion (37–39) may explain the significant improvement in physical interference among the vitamin D + physiotherapy group patients.

Our study suggested that the patients with LBP, neck pain, shoulder pain, and knee pain were equally benefited from vitamin D supplementation along with physiotherapy. Managing one of the major global burdens, that is, LBP is a significant challenge for the clinicians (40). Our study showed that supplementation of vitamin D along with physiotherapy provides better efficacy compared to physiotherapy alone when managing LBP. A systematic review and meta-analysis published in 2017 concluded that there is an inconsistent association between vitamin D deficiency and LBP (8). Similarly, another systematic review of clinical trials indicated that vitamin D supplementation was not more effective than placebo in LBP (41). Evidence from our study provides a scientific clue to use vitamin D supplementation as a combined therapy when managing LBP for best results. Additional randomized control trials are warranted to conclude this result.

A systematic review has revealed the beneficial effect of vitamin D supplementation when treating neck pain (10). Similarly, a higher prevalence of hypovitaminosis D has been found in patients with knee and shoulder pain (9, 11, 42). Additional randomized control trials are required to further evaluate the effect of vitamin D supplementation among patients with neck, shoulder, and knee pain.

The association between hypovitaminosis D and poor health outcome is well-known; however, the relationship between vitamin D and chronic pain is not clear yet. Furthermore, the mechanism by which vitamin D inhibits chronic pain development is still ambiguous. In light of the current lack of evidence, supplementation of vitamin D cannot be recommended as an effective independent treatment for chronic pain (43). However, for better treatment of chronic pain, vitamin D in combination with other treatments is beneficial (44). In our study, we found better efficacy in chronic pain management when vitamin D was used with physiotherapy. On the other hand, limited information is available regarding the effectiveness of vitamin D for acute and sub-acute pain management; however, our study found similar efficacy for chronic pain management when treated with vitamin D and physiotherapy.

### Strength of the Study

The strength of the study includes (1) sufficient number of patients recruited for both the vitamin D + physiotherapy and physiotherapy groups; (2) equal distribution of patients for each sub-group, for example, sex, age group, chief complaints, and pain duration; and (3) the standardized collection of data and pathological tests from a single research center.

### Limitations of the Study

There were three limitations of this study. First, our study's quasi-experimental methodology was less reliable than a randomized controlled trial. Second, we included patients with the combination of vitamin D and physiotherapy treatment for vitamin D deficient patients, which may have influenced the pain score. Third, because of cost constraints, vitamin D concentration from blood was not tested after 12 weeks of intervention in this study.

In conclusion, this study gives information on the future possible use of vitamin D supplementation in the treatment of patients with MSD when used in conjunction with physiotherapy. Combined intervention (vitamin D with physiotherapy) showed significantly better results than only physiotherapy intervention in the reduction of three pain-related outcomes, pain severity, affective interference, and physical interference, in musculoskeletal pain. To confirm these findings, more research is needed, including randomized controlled trials with placebo controls and longer follow-ups.

## Data Availability Statement

The raw data supporting the conclusions of this article will be made available by the authors, without undue reservation.

## Ethics Statement

The studies involving human participants were reviewed and approved by Ethical Review Committee (ERC) of Uttara Adhunik Medical College and Hospital. The patients/participants provided their written informed consent to participate in this study.

## Author Contributions

MA conceived the idea, designed and conducted the trial, managed the project and participants, interpreted the data, drafted, and review the manuscript. ZU and AH supervised the study and review the manuscript. MA and AH performed the statistical analysis. All authors contributed to the article and approved the submitted version.

## Conflict of Interest

The authors declare that the research was conducted in the absence of any commercial or financial relationships that could be construed as a potential conflict of interest.

## Publisher's Note

All claims expressed in this article are solely those of the authors and do not necessarily represent those of their affiliated organizations, or those of the publisher, the editors and the reviewers. Any product that may be evaluated in this article, or claim that may be made by its manufacturer, is not guaranteed or endorsed by the publisher.

## Abbreviations

MSD: musculoskeletal disorders
LBP: low back pain
BPI: Brief Pain Inventory.
